# Fine tuning breath‐hold‐based cerebrovascular reactivity analysis models

**DOI:** 10.1002/brb3.426

**Published:** 2016-01-25

**Authors:** Christiaan Hendrik Bas van Niftrik, Marco Piccirelli, Oliver Bozinov, Athina Pangalu, Antonios Valavanis, Luca Regli, Jorn Fierstra

**Affiliations:** ^1^ Department of NeurosurgeryUniversity Hospital ZurichUniversity of ZurichClinical Neuroscience CenterFrauenklinikstrasse 108091ZurichSwitzerland; ^2^ Department of NeuroradiologyUniversity Hospital ZurichUniversity of ZurichClinical Neuroscience CenterFrauenklinikstrasse 108091ZurichSwitzerland

**Keywords:** BOLD, breath‐hold, cerebrovascular reactivity, human brain, hypercapnia

## Abstract

**Introduction:**

We elaborate on existing analysis methods for breath‐hold (BH)‐derived cerebrovascular reactivity (CVR) measurements and describe novel insights and models toward more exact CVR interpretation.

**Methods:**

Five blood‐oxygen‐level‐dependent (BOLD) fMRI datasets of neurovascular patients with unilateral hemispheric hemodynamic impairment were used to test various BH CVR analysis methods. Temporal lag (phase), percent BOLD signal change (CVR), and explained variance (coherence) maps were calculated using three different sine models and two novel “Optimal Signal” model‐free methods based on the unaffected hemisphere and the sagittal sinus fMRI signal time series, respectively.

**Results:**

All models showed significant differences in CVR and coherence between the affected—hemodynamic impaired—and unaffected hemisphere. Voxel‐wise phase determination significantly increases CVR (0.60 ± 0.18 vs. 0.82 ± 0.27; *P* < 0.05). Incorporating different durations of breath hold and resting period in one sine model (two‐task) did increase coherence in the unaffected hemisphere, as well as eliminating negative phase commonly obtained by one‐task frequency models. The novel model‐free “optimal signal” methods both explained the BOLD MR data similar to the two task sine model.

**Conclusions:**

Our CVR analysis demonstrates an improved CVR and coherence after implementation of voxel‐wise phase and frequency adjustment. The novel “optimal signal” methods provide a robust and feasible alternative to the sine models, as both are model‐free and independent of compliance. Here, the sagittal sinus model may be advantageous, as it is independent of hemispheric CVR impairment.

## Introduction

Cerebrovascular reactivity (CVR) indicates remaining reserve capacity of the cerebrovascular autoregulation to maintain cerebral blood flow (CBF) (Sobczyk et al. 2014). Measuring CVR variations within the brain has the potential to detect cerebrovascular pathophysiology (Fierstra et al. 2013; Willie et al. 2014) such as increased stroke risk, neuropsychological deficits(Marshall and Lazar 2011) and neurostructural changes in the absence of acute ischemia (Conklin et al. 2010; Fierstra et al. 2010). It can be measured by applying a universal vasoactive stimulus, for example, carbon dioxide (CO_2_) increases via breath‐holding (BH), and measure the subsequent cerebrovascular response. For example, at our institution we employ functional intraoperative blood‐oxygen‐level‐dependent magnetic resonance imaging (BOLD fMRI) measurements with three cycles of apnea as a vasoactive stimulus (mimicking expiratory BH) in mechanically ventilated neurosurgical patients.

BH is a clinically inexpensive and straightforward method with BOLD changes arising after as soon as 3 sec (Abbott et al. 2005). It remains challenging, however, to accurately interpret BOLD response to BH. BH is usually repeatedly performed at end‐expiration lasting for approximately 20–30 sec, by which ongoing oxidative metabolism and restricted respiratory outwash results in accumulation of arterial partial pressure of CO_2_ (PaCO_2_) (Ratnatunga and Adiseshiah 1990). In the healthy brain, the following CO_2_‐related CBF increase results in more oxyhemoglobin with subsequent higher BOLD signal (Kastrup et al. 1999). Longer BH durations may ensure a more robust physiological response (Lui et al. 2002; Magon et al. 2009; Bright and Murphy 2013) but often cannot be maintained by subjects. Consequently, intra‐ and intersubject CVR results become increasingly variable with shorter BH duration and bad compliance.

Since BH results in a gradual PaCO_2_ increase (Sasse et al. 1996) Murphy et al. (2011), advocated the need to adjust the boxcar function regressor to better explain the dynamic response of the BOLD signal during BH. The authors proposed the use of either a sine wave at the task frequency or end tidal CO_2_ (PETCO_2_) tracer convolved with the hemodynamic response function and applied a single global phase shift to further improve these regressors. Although both regressor models show good results, the application of a single global time lag for the entire brain inherently limits the interpretation of temporal CO_2_‐related BOLD signal changes between different regions of the brain (Rostrup et al. 2000; Andrade et al. 2006; Bright et al. 2009).

This is a potential limitation in patients with cerebrovascular pathology, where regional BOLD signal changes are even more inhomogeneous due to reduced or delayed arrival of blood to the brain, thereby altering the accuracy of CVR interpretation.

Geranmayeh et al. (2015) created a better fit of BOLD data by combining PETCO_2_ monitoring during BH with a voxel‐wise time lag. PETCO_2_ tracing in the clinical setting may impose some limitations, however, such as the need for extra equipment and often insertion of a nasal cannula requiring subjects to breathe through their nose or placement of a facial mask thereby limiting comfort. Therefore, a BH timing independent or “optimal signal” method was used as an alternative analysis strategy. Here, the mean time series of the contralateral—unaffected—hemisphere was taken as a regressor, increasing the explained variance compared to the voxel per voxel analysis of PETCO_2_ BH. Nevertheless, the use of the contralateral hemisphere has a significant limitation: CVR impairment frequently transcends beyond the affected hemisphere, and may also be present in the contralateral ′′unaffected′′ hemisphere (Sam et al. 2014). Merging the mean unaffected hemisphere time series will therefore result in erroneous CVR interpretation.

The present work elaborates on the aforementioned analysis models to increase sensitivity and reproducibility of BH derived BOLD fMRI CVR measurements. The approach is threefold: First, we assess the differences between the sine regressor with a single universal brain lag (global‐delay sine) as proposed by Murphy et al. (2011) and the same model with a voxel‐wise lag (voxel‐wise delay sine) in subjects with unilateral hemodynamic impairment. Secondly, we propose a parametric variant of the sine model. Earlier sine models encompassed one task frequency although BH and resting periods could have an amply different duration. We have therefore created a combined model of two task frequencies to compensate for different BH and resting duration, aiming to create a better fit to the BOLD data.

Finally, to further improve CVR maps reliability, we modeled subject‐specific response to BH by a data based, physiologically optimal, and behavior consistent regressor for each subject's particular response to the given stimulus. While combining positive and negative time series can potentially neutralize mean time series of the hemisphere, we used the time series of the sagittal sinus—the brain's major venous outflow. In theory this should describe the most robust patient specific cerebral positive BOLD response (i.e., collection point of oxyhemoglobin in a concentric anatomical structure), least influenced by potential negative CVR alterations (Pillai and Mikulis 2015). This “sagittal sinus” model was compared to the “Unaffected” Hemisphere model.

## Methods

### Clinical datasets

Datasets of five neurosurgical subjects exhibiting unilateral hemispheric impaired perfusion, as diagnosed with H_2_O‐Positron Emission Tomography (PET) imaging, were taken from an ongoing prospective study of intraoperative BOLD CVR measurements at the Department of Neurosurgery, University Hospital Zurich, Switzerland, approved by the cantonal ethics board of the Canton of Zurich, Switzerland (KEK‐ZH‐Nr. 2012‐0427).

### MRI protocol

MRI data were acquired on a 3‐Tesla Skyra VD13 (Siemens, Erlangen, Germany) with an ellipse‐shaped intraoperative 8 channels head coil (NORAS MRI products, Hochberg, Germany). Whole‐brain 2D BOLD fMRI EPI (Mansfield 1977) sequence planned axially on the anterior commissure—posterior commissure (ACPC) line plus 20° on a sagittal image with voxel size: 3 × 3 × 3 mm^3^, acquisition of matrix 64 × 64, 35 slices with ascending acquisition, slice gap 0.3 mm, GRAPPA factor 2 with 32 ref. lines, adaptive Coil Combination, Auto Coil Selection, Repetition Time (TR)/TE 2000/30 ms, flip angle 85° (optimized for gray matter), bandwidth 2368 Hz/Px, 220 volumes, Field of View 192 × 192 mm. Furthermore, a 3D T1‐weighted MPRAge image was also planned on the ACPC line on a sagittal image with recon voxel size: 0.47 × 0.47 × 0.9 mm^3^, acquisition of matrix 256 × 256, 192 slices, slice oversampling 25%, GRAPPA factor 2 with 24 ref. lines, adaptive Coil Combination, TR/TE/TI 1900/2.60/900 ms, flip angle 9°, bandwidth 220 Hz/Px, Field of View 240 × 240 mm. The T1‐image was acquired for coregistration, skull stripping, and overlay purposes.

### Breath hold paradigm

**Figure 1:**
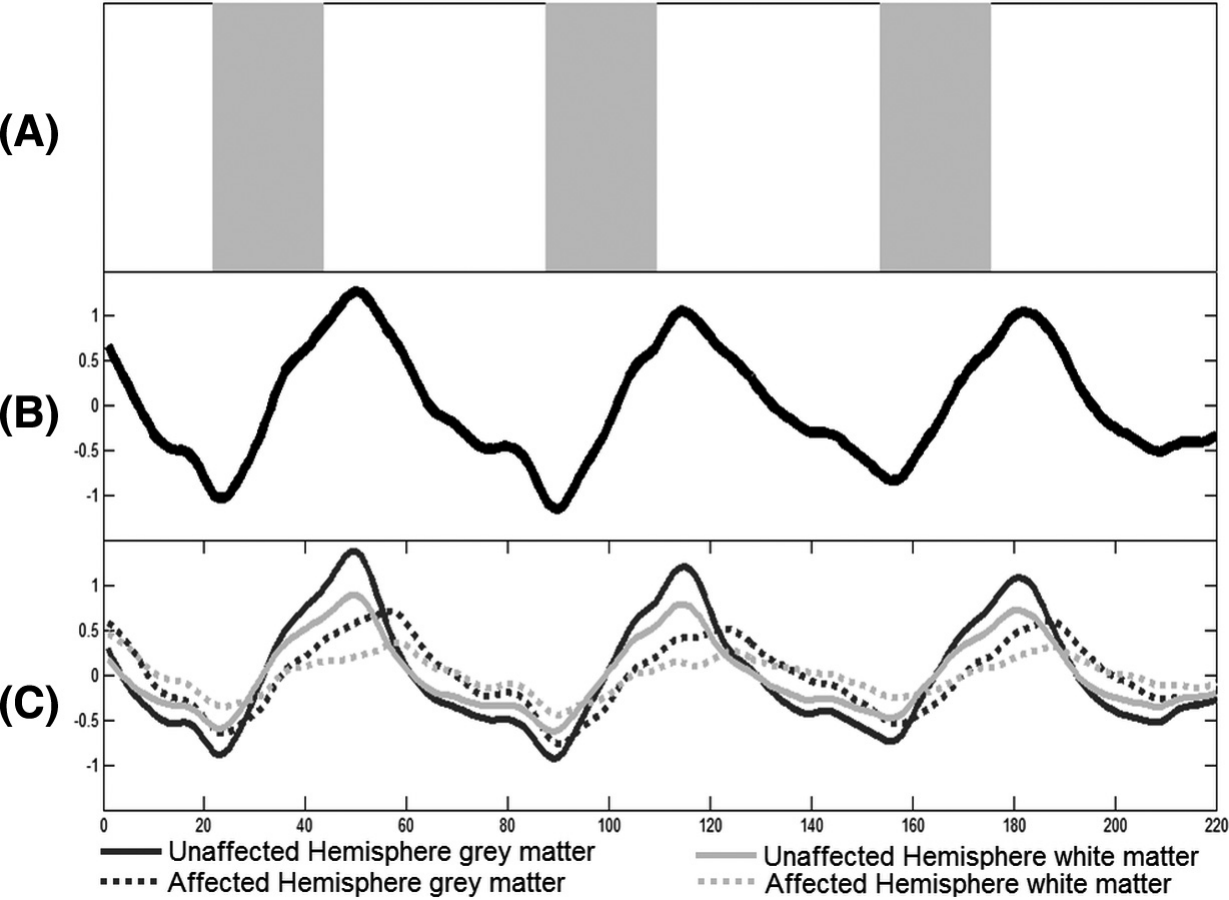
Breath‐hold paradigm and BOLD time series. (A) Breath‐hold paradigm presented as a taskbar. The gray blocks indicate the three breath hold periods of 22 TR (44 sec). The white blocks, amidst, represent the resting periods of 44 TR each, except for the first one: 22 sec. Total duration of this protocol is 7:20 min. (B) Illustrative whole brain combined gray and white matter BOLD time series of one subject. The time series is displayed over the period of 220 repetition times (TR) (440 sec). (C) Hemispheric mean BOLD time series of gray and white matter. The time courses of the unaffected hemisphere are illustrated with a bold line, whereas the affected hemisphere time courses are displayed with a dotted line. The dark lines indicate gray matter, whereas gray lines indicate the white matter. As expected, the unaffected hemisphere reacts to a greater extent to the given stimulus than the affected hemisphere, as does the gray matter compared to the white matter. The time to reach signal maximum is also faster in the unaffected hemisphere, which results in different phase between hemispheres.

The BH paradigm consisted of a 44 second preparation period of ventilator controlled breathing after which apnea was induced for 44 sec by turning off the ventilator. After the BH period, the ventilator controlled breathing was resumed. To simulate post‐BH hyperventilation, we applied a series of six manual ventilations with an AMBI bag to facilitate apt return to baseline CO_2_. In total, three repetitive cycles with a BH block of 44 sec and following resting baseline block of 88 sec were performed (Fig. 1A).

### Data analysis

#### Spatial preprocessing

Imported anatomical and functional images were spatially preprocessed using Statistical Parameter Mapping 12 (SPM 12, Wellcome Trust Centre for Neuroimaging, Institute of Neurology, University College London, UK; http://www.fil.ion.ucl.ac.uk/spm/). Due to the four head point fixation of the subject in MRI and f0 drift monitoring, realignment of BOLD data has shown unnecessary and only a mean BOLD volume was calculated. The MPRAge T1 image was coregistered to the mean BOLD image and automated segmentation was performed, resulting in gray matter, white matter, cerebrospinal fluid, skull, skin, and air probability maps. The functional images were spatially smoothed with an isotropic Gaussian kernel of 8 mm full‐width at half maximum. Last, the anatomical hemispheres were manually segmented in left and right hemisphere.

#### Temporal preprocessing

After preprocessing, the MRI data were further analyzed with an in‐house script developed on MATLAB R2013b (The MathWorks, Inc., Natick, Massachusetts; http://www.mathworks.com/). A low pass filter with a filter cut‐off frequency of 0.125 Hz —chosen based on figure 2 in Duffin et al. (2015)—was applied to the MR data. MR time courses were normalized by the mean signal and detrended by a linear fit to the data to correct for MR signal drift, presenting data as percent BOLD. Last, the BOLD time series were temporally smoothed by 14 (6%) dynamic local regression using weighted linear least squares and a second polynomial model with assignment of lower weight to outliers (the so‐called robust Loess method).

### Global versus voxel‐per‐voxel time lag

**Figure 2:**
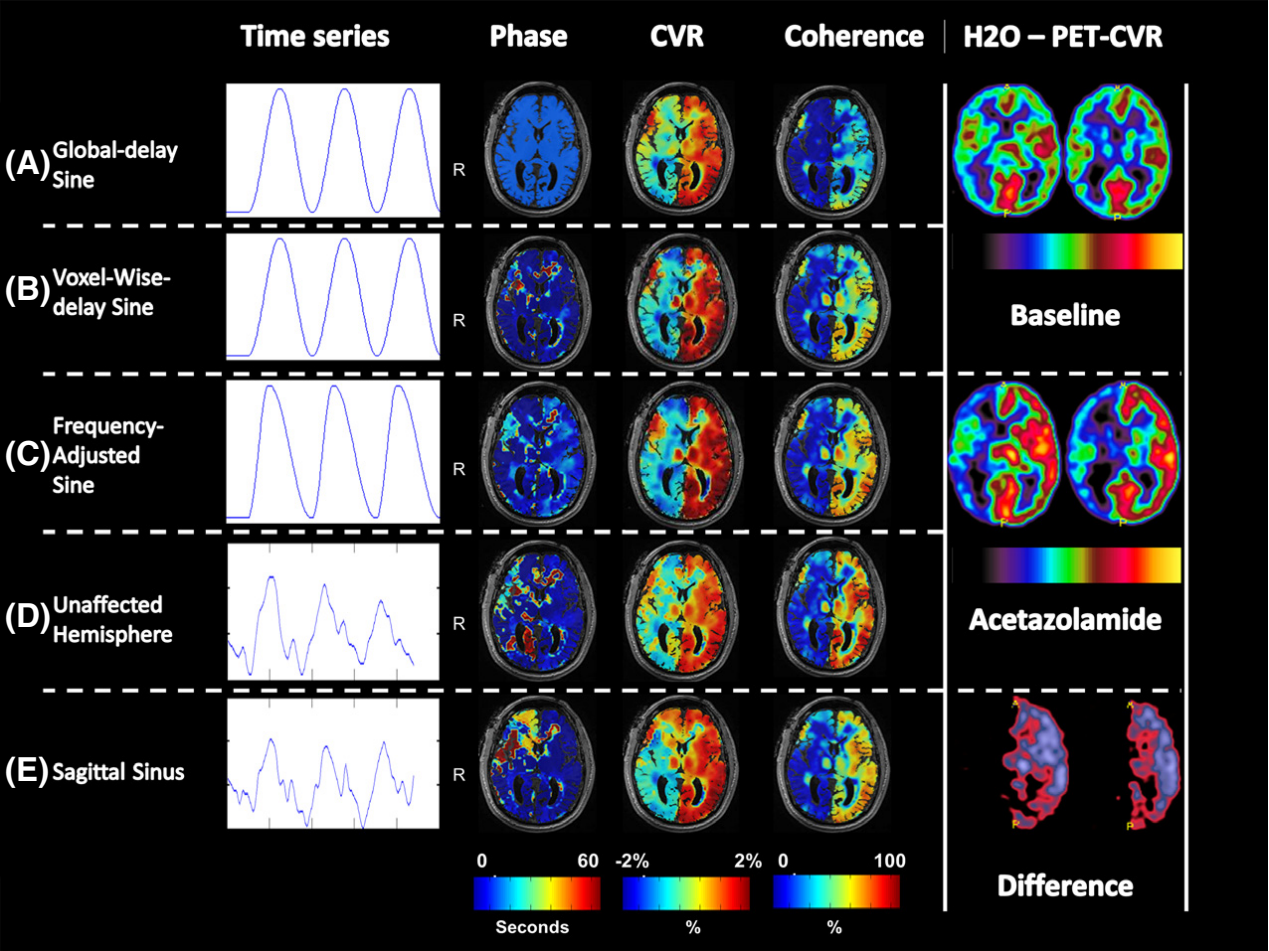
Illustrative models and phase, CVR, and coherence maps of one subject (male: 65 years old) with right ICA occlusion and right‐sided unilateral hemodynamic impairment on H_2_O‐PET imaging. The color scales are displayed below the maps. For the phase maps, the scale ranges from 0 to 60 sec. The CVR maps of the sine models display the % BOLD signal change and are ranged from −2 and 2%, whereas the “Optimal Signal” models represent the % BOLD signal change per percent optimal BOLD time series and are also ranged from −2 to 2%. The coherence maps show the measurement of the amount of variance explained with the given model between 0–1. Abbreviations: CVR, cerebrovascular reactivity; ICA, internal carotid artery; PET, positron emission tomography; TR, repetition time.

To differentiate between CVR analysis with a single global delay and a voxel‐wise delay, we created a sinusoid regressor analogously to Murphy et al. (2011):

- Sine with the task frequency of f = 1/132 Hz and a phase shift (Fig. 2A+B).

We implemented a best fit (maximum Pearson correlation coefficient with L2‐norm) of this sine regressor to the gray and white matter average BOLD signal time course (“global‐delay sine”) and of each voxel BOLD time series (“voxel‐wise‐delay sine”). Phase was defined as the model time shift, determined with maximum Pearson correlation coefficient, combining the arrival time of CO_2_ and, partially, the dynamic response of that voxel. The better the model fits the BOLD time course, the more does the phase reflect the real CO_2_ arrival time. To obtain a meaningful phase model, the correlation calculation was limited to a lower bound of −11 TR (TR = 2 sec) and an upper bound of 30 TR. The lower limit was chosen to compensate for the fact that fast responding voxels could peak at 44 sec (i.e., end of the first BH period), whereas the regressor reaches the first peak at 55 TR. The upper correlation threshold of 30 TR was applied to reduce overfitting of the data to noise. After 30 TR, we determined the phase to be physiological highly improbable. Subsequently, CVR was determined as the slope of the linear least square fit of the models against the BOLD signal time course. Finally, *R* ^2^ (coherence), defined as the amount of variance explained by the given model, was calculated for every voxel.

After application of a combined gray and white matter binary mask (total probability threshold of 0.9), coherence of each model was compared for whole brain and both hemispheres separately. Voxels with a coherence of less than 0.05 were considered not explained by the model. The percentage of voxels surpassing the threshold “coherence >0.05” was calculated with the following equation:

$$\%\text{Significant Voxels}=\frac{\text{Voxel explained}}{\text{voxel total}}\times 100$$

where % significant voxels is the percentage of voxels surpassing the 0.05 threshold, Voxelexplained is the amount of remaining voxels after coherence thresholding, and Voxeltotal is the total amount of voxels after masking with gray and white matter probability maps. An independent two sample *T*‐test was used to determine potential significant differences in phase, CVR and coherence across models, where the paired *T*‐test was used to reveal potential differences between hemispheres of the same model. If no significance was found, a difference analysis (h≠0) with a one sample *t*‐test was performed to show significant change in CVR or coherence by a model or hemisphere. To determine a potential relationship between phase and CVR, a linear regression of averaged hemispheric phase and CVR calculations was performed to determine *R* ^2^.

### Parametric frequency‐adjusted sine

The abovementioned sine models are based on one task frequency even though the BH periods and ventilation periods are not similar in duration. We assume that these models break down the longer the ventilation periods gets relative to the BH period. Therefore, we adjusted the sine with a separate task frequency for the BH period (*f* = 1/44 Hz) and for the resting period (*f* = 1/88 Hz) to create the new model: “frequency‐adjusted sine” (Fig. 2C). Phase, CVR, and coherence were calculated similar to the other models (global sine and voxel‐wise‐delay sine). The ranges for the phase calculations were similar to the voxel‐wise‐delay sine. Again the relationship between phase and CVR was calculated.

### Novel “Optimal Signal” models

While the mean over the hemisphere would potentially be neutralized by combining positive and negative time series, the collection of oxyhemoglobin in the sagittal sinus close to the confluence of sinuses would result in voxels with an optimal signal response for the given paradigm. On the T1 image, approximately 10 voxels within the center of the confluence of sinuses or inferior part of the superior sagittal sinus were used to create a mask and subsequently applied to the functional MRI data. The mean time course over these voxels, identified as the brain venous output BOLD time course for that study protocol, was used to compute the sagittal sinus model (Sagittal Sinus—Fig. 2E). The Pearson correlation was also estimated for this model up to a delay threshold of 30 TR. Contrary to the other models, this method resulted in a combined arterial/venous phase map (Fig. 2E). Therefore, negative delays up to 5 TR were accepted accounting for any venous phase of fast responding voxels.

Furthermore, this model was compared to the combined gray and white matter time series of the contralateral (unaffected) hemisphere (“Unaffected Hemisphere” model; Fig. 2D).

The CVR was calculated relative to the BOLD signal in the sagittal sinus or mean unaffected hemisphere and was therefore stimulus independent.

## Results

The mean phase, CVR, and coherence of each regressor for whole brain and unaffected and affected hemisphere are presented in Table 1. Observing the mean time series over the whole brain and both hemispheres separately, no signal plateau could be identified even after 44 sec of BH (Fig. 1). However, looking at gray and white matter separately, some datasets showed a gray matter signal plateau in the hemisphere with hemodynamic impairment.

**Table 1:** Phase, CVR, and Coherence defined by whole brain and hemispheres

		Whole brain	Unaffected hemisphere	Affected hemisphere
Global‐delay sine	Phase	6.40 ± 9.53	6.40 ± 9.53	6.40 ± 9.53
	CVR	0.60 ± 0.18	0.88 ± 0.23	0.36 ± 0.24*
	Coherence	23 ± 10	26 ± 10	20 ± 11*
Voxel‐wise‐delay sine	Phase	1.60 ± 5.55	−2.2 ± 4.60	8 ± 4.47*
	CVR	0.82 ± 0.27	1.38 ± 0.28	0.40 ± 0.24*
	Coherence	41 ± 15	49 ± 15	35 ± 17*
Frequency‐adjusted sine	Phase	11 ± 2.32	6.75 ± 3.26	14.72 ± 4.32*
	CVR	0.97 ± 0.25	1.61 ± 0.29	0.43 ± 0.24*
	Coherence	44 ± 17	53 ± 16	37 ± 18*
Unaffected hemisphere	Phase	10.20 ± 6.92	3 ± 2.82	15.20 ± 9.54*
	CVR	0.74 ± 0.21	1.07 ± 0.14	0.46 ± 0.37*
	Coherence	52 ± 20	63 ± 20	42 ± 21*
Sagittal sinus	Phase	11.60 ± 5.72	2.20 ± 5.22	18 ± 8.12*
	CVR (%)	1.21 ± 1.06	1.82 ± 1.81	0.64 ± 55
	Coherence	47 ± 19	56 ± 19	39 ± 21*

All phase calculations are in seconds. CVR is presented as % ΔBOLD. Coherence is % explained variance.

Mean ± SD.

*P < 0.05 comparing affected and unaffected hemisphere.

### Global‐delay sine versus voxel‐wise‐delay sine

Illustrative high‐resolution phase, CVR, coherence, and H_2_O PET‐CVR maps of one subject are shown in Figure 2. The H_2_O PET image shows a unilateral CVR impairment with normal contralateral CVR. At first glance, the maps in Figure 2 show a high visual correlation of phase, CVR, and coherence between models, but in detail the maps differ substantially. For instance, the high‐resolution maps show the regional benefits of implementing a voxel‐wise phase (Fig. 2). In global‐delay sine, the use of one shift for the whole brain results in areas with impaired CVR (green color: near zero). The implementation of a voxel‐wise phase results in paradoxical CVR (blue color: negative) with a higher coherence in those regions.

Averaged over the whole brain, there was no significant difference between the phase of the global shift and the voxel‐wise‐delay shift, whereas the voxel‐wise‐delay shift was significantly different when hemispheres were analyzed separately. Parts of the affected hemisphere showed a higher phase as can also be appreciated in Figure 2. Both sine models showed significant greater CVR in the unaffected hemisphere than the affected hemispheres.

Significant difference in CVR was also found between global‐delay and voxel‐wise sine for the unaffected hemisphere (*P* < 0.05), although this was not found for the affected hemisphere.

**Figure 3:**
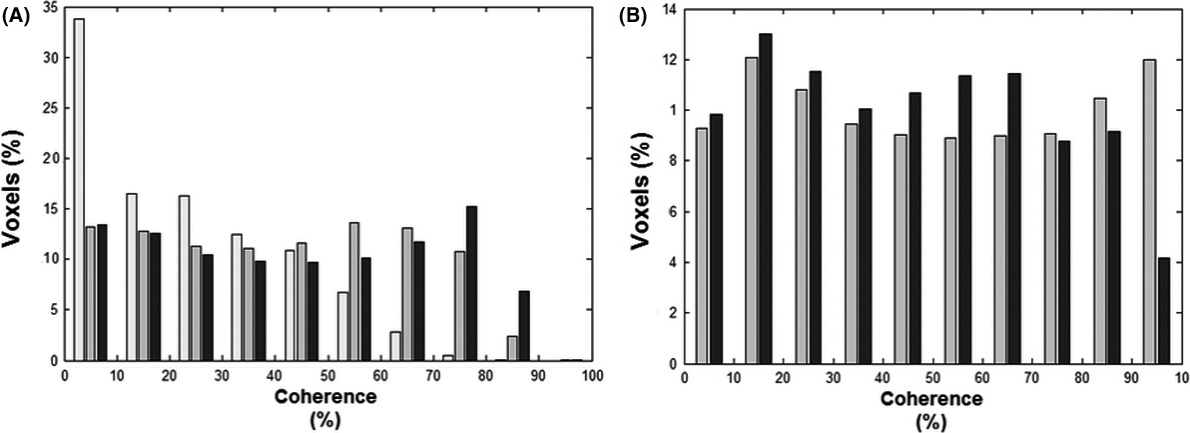
Coherence histograms of five analysis models. To evaluate the effectiveness of each model, the mean percent coherence per bins of 10% coherence was determined and presented in two histograms. (A) The histogram portrays the number of voxels explained by the three sine models. From left to right: global‐delay sine (white bars), voxel‐wise‐delay sine (gray bars), and frequency‐Adjusted sine (black bars). The global‐delay sine explains almost a factor of three more voxels with coherence below 10% and is decreasingly present in bins with higher coherence. Compared to the voxel‐wise sine, the frequency‐adjusted sine is only more present in the bins from 70% and higher. (B) The histogram portrays the number of voxels explained by the two “Optimal Signal” models divided in bins of 10% coherence. The unaffected hemisphere (gray bars) has a high number of voxels explained in the bins between 70 and 100%, whereas the sagittal sinus (black bars) explains more voxels in de lower bins.

**Figure 4:**
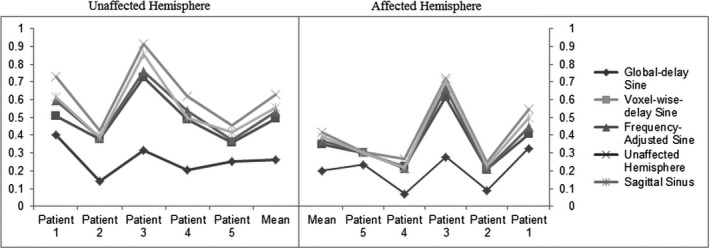
Coherence of the affected and unaffected hemisphere. Coherence of three sine models and two optimal signal models are displayed per subject and as a mean over the whole group for the unaffected hemisphere (left panel) as the affected hemisphere (right panel). All models showed a significant difference between the both hemispheres (*P* < 0.05). For the sine models, coherence in the unaffected hemisphere increased significant with the implementation of a voxel‐wise delay. Difference analysis showed a significant positive increase in the affected hemisphere after implementation of a voxel‐wise phase. Frequency‐adjusted sine shows only in the unaffected hemisphere a significant increase in coherence after difference analysis. A significant coherence difference between the sagittal sinus and unaffected hemisphere was found for both hemispheres (*P* < 0.05).

**Figure 5:**
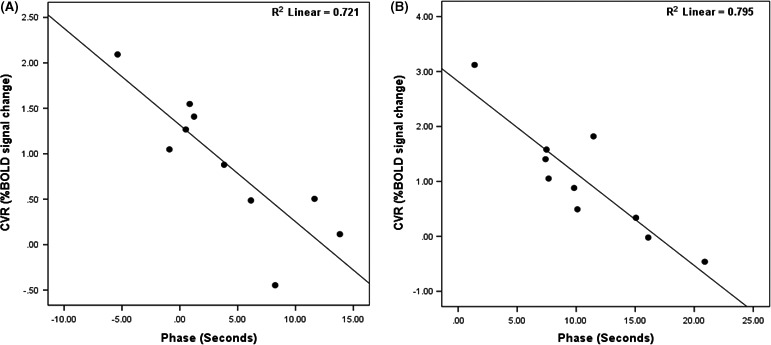
Hemispheric average phase—CVR Plot. Hemispheric average phase plotted against hemispheric average CVR for both the voxel‐wise‐sine (A) as for the frequency‐adjusted sine (B). (A) (*R* ^2 ^= 0.721, *P* < 0.001) and (B) (*R* ^2 ^= 0.795, *P* < 0.001) both show good correlation with a negative slope, meaning longer phase correlated with more impaired CVR. CVR, Cerebrovascular reactivity.

After application of the coherence threshold of 0.05, the models with a voxel‐wise shift showed a significant increase in the percent of voxels surpassing the threshold, compared to the global sinus (*P* < 0.05). This global coherence difference divided in bins of 10% can be observed in Figure 3. An obvious difference can be seen between the global‐delay sine and voxel‐wise sine models. The coherence of the affected and unaffected hemisphere is presented in Figure 4. A significant difference in coherence in the unaffected hemisphere between the voxel‐wise‐delay sine and the global‐delay sine was found (*P* < 0.05). The whole brain and affected hemisphere coherence of the global‐delay sine were not significantly lower than the voxel‐wise‐delay sine, but after a one sample *t*‐test analysis on the subtracted difference both show a significant increase (whole brain: *P* = 0.02, unaffected hemisphere: *P* = 0.03) For the voxel‐wise‐delay sine, a linear regression was performed to find the correlation between average phase and CVR over both hemispheres, shown in Figure 5A. Taken the average phase and CVR of both hemispheres of each subject into account, phase shows a good correlation with CVR (*n* = 10, *R* ^2 ^= 0.721, *P* < 0.001).

### Frequency‐adjusted sine

For the second analysis, we compared a sine model, combining two task frequencies for the BH and resting period (frequency‐adjusted sine), with the sine model with 1 task frequency (voxel‐wise‐delay sine). With the 1 task frequency sine model, a high number of voxels with negative phase (24421 ± 77840 – 51% ± 14) were found. This phenomenon can be explained by observing gray and white matter time courses depicted in Figure 1. Here, both time courses of the healthy hemisphere reach their first peak before 55 TR (see Methods). Calculating the Pearson maximum correlation with the 1 task frequency sine model will result in negative phase for these time courses. No significance difference in CVR was found between both models (*P* > 0.05). Similar to CVR, a comparable coherence is observed between voxel‐wise‐delay sine and the frequency‐adjusted sine (*P* > 0.05) over the whole brain (see Table 1 and Fig. 4). However, after analysis of the difference between means for the 1 and 2 task frequency models, a significant increase in coherence for the unaffected hemisphere was found (*P* < 0.05).

Similar to the voxel‐wise‐delay sine, a good correlation was found between phase and CVR (*n* = 10, R = 0.795, *P* < 0.001; Fig. 5).

### Novel “Optimal Signal” models

Finally, we created two data derived model‐free “Optimal Signal” methods out of voxels within (1) the Confluence of Sinuses (sagittal sinus) and (2) of the unaffected hemisphere (Unaffected Hemisphere). Illustrative time courses and phase, CVR, and coherence maps of both models are shown in Figure 2. Both Optimal Signal models have a significantly greater CVR as compared to all sine models. Significant CVR differences can be seen between the unaffected and affected hemisphere for the Unaffected Hemisphere model but not for the sagittal sinus model. Though, the difference analysis showed a clear significant difference between hemispheres. (*P* < 0.05) The sagittal sinus model shows a factor 3 greater CVR between the unaffected versus the affected hemisphere. The Unaffected Hemisphere model shows only a factor 2 difference between hemispheric CVR. No significant difference in CVR for either hemisphere was found between both Optimal Signal models. Both models fit more voxels with a coherence higher than 0.05 (Fig. 3), although a significant difference was only found between the sine models and the sagittal sinus (*P* < 0.05).

As for coherence, both models show a significant difference between the affected and unaffected hemisphere (*P* < 0.05). Using an independent two sample *T*‐test, there was no statistical difference in coherence between Unaffected Hemisphere and sagittal sinus (*P* = 0.7153), nevertheless difference analysis showed a significant increase in coherence for both hemispheres in favor of the Unaffected Hemisphere. Only the Unaffected Hemisphere showed a significant positive difference between the frequency adjusted sine model after difference analysis (unaffected hemisphere: *P* = 0.009; affected hemisphere: *P* = 0.04) As an example, as shown in [Link], one extra BH dataset from a subject with an asymptomatic bilateral internal carotid artery occlusion is presented. In that subject, no clear “unaffected hemisphere” can be found.

## Discussion

With this work we elaborate on previous analysis models proposed by Murphy et al. (2011) and Geranmayeh et al. (2015), to further improve CVR analysis methods derived from BOLD fMRI BH datasets. Specifically, we optimized the sine analysis model with 1 task frequency by adding voxel‐wise phase and generalized it to different durations of BH and resting periods, in order to calculate percent BOLD signal change (CVR) and, thereby, generating better explained variance (coherence). Additionally, we verified our novel “Optimal Signal” model by comparing it to the unaffected hemisphere model introduced by Geranmayeh et al. (2015).

Regarding the sine models, the phase consists of the temporal lag between the sine model and the corresponding dynamic response of the vessels. If the model better describes the dynamic vessel responses (i.e., by high coherence), the absolute phase will be smaller and will better reflect the true arterial temporal lag. After optimizing the phase for maximal correlation, the subsequent CVR, defined as percent BOLD signal change, states the relative response of the voxel, whereas the coherence explains how well the model fits the dynamic response. For all models we showed a high discrepancy in CVR for the unaffected and affected hemisphere. This indicates a robust stimulus with subsequent BOLD signal change to determine brain areas with normal CVR, impaired CVR or even steal phenomenon—that is, paradoxical CVR (Sobczyk et al. 2014).

### Global‐delay sine versus voxel‐wise delay sine

Our finding of a global phase of 6.4 ± 9.53 showed a good correlation with findings by others (Panerai et al. 2000; Wise et al. 2004; Murphy et al. 2011). We increased sensitivity by implementing voxel‐wise phase to a sinus regressor with one task frequency. Our results clearly confirm that using a voxel‐wise phase results in a statistically significant increase in CVR with the given model. Improvement of a voxel‐wise shift can also be seen in the difference between the two hemispheres. Phase is significantly different between hemispheres for the voxel‐wise‐delay sine stating that the phase is a confounder for CVR determination. By calculating the linear regression between the average phase and CVR found in the unaffected and affected hemisphere, we did find a good agreement between phase and CVR for both voxel‐wise sine models (*R* ^2 ^= 0.721 vs. *R* ^2 ^= 0.795), with a negative slope indicating lower CVR with longer phase. This corresponds with findings by Poublanc et al. (2013) showing longer bolus arrival times (as determined on Dynamic Susceptibility Contrast‐enhanced MRI) with more impaired CVR and a good correlation between increasing phase and bolus arrival time. On the contrary, negative phase did not correlated well with bolus arrival time. However, in theory, negative phase should not be possible based on their method employing continuous end‐tidal CO_2_ tracing, as it is physiologically unlikely for the BOLD signal to react before CO_2_ changes are apparent. Phase in the affected hemisphere is not only longer due to increasing temporal lag but also due to a different vascular response delay (Fig. 1). This vascular response consists of the CO_2_ receptor sensitivity and can differ within clinical populations and between age ranges (Taoka et al. 1998; Thomason et al. 2005; Handwerker et al. 2007; Chang et al. 2013). This is also reflected by coherence and the percentage of significant voxels for a coherence threshold of 0.05. A significantly higher percentage voxels explained by the voxel‐wise‐delay sine indicates a better fit to the data.

That the statistical difference of the coherence is only seen in the healthy hemisphere, states that implementation of a voxel‐wise shift for sine with 1 task frequency results mainly in a better verification of healthy time series. This opens up the need to define a different model in the affected hemisphere.

From our data, we are able to explain a similar amount of voxels with a coherence higher than 0.05 with our sine models as Geranmayeh et al. (2015) did for the end tidal CO_2_ tracer convolved with the HRF for a global‐ and a voxel‐wise phase.

Globally, these results are in line with previous findings stating that the sine regressor explains a similar amount of variance as CO_2_ tracer (Murphy et al. 2011; Lipp et al. 2015). Our results, however, did show a much higher mean explained variance over the affected hemisphere. This can be explained by a 14 sec longer BH duration. But potentially of more relevance is the implementation of a better spatiotemporal filtering, removing noise thereby increasing coherence. Interestingly, in the intraoperative setting, we did not observe the presence of a signal plateau in the healthy hemisphere —after 44 sec of BH. However, the gray matter of the affected hemisphere did show a plateau in some datasets. Finding a signal plateau for healthy tissue would have resulted in determining an optimal BH paradigm. Our BH paradigm was based on TR's. Therefore, starting the BH period was not based on inspiratory or expiratory exercise. However, in the intraoperative setting, higher pressures are needed to ventilate a subject. Shutting down the ventilator will result in a last expiratory breathing motion and thus intraoperative BH can be compared to expiratory BH. Lui et al. (2002) showed the appearance of a BH plateau of the amount of significantly activated voxels after 20 sec of expiratory BH. However, they did not show the presence of a definitive BOLD signal plateau. That we were not able to find a signal plateau for the unaffected hemisphere could be a dampened response, attributed to the intraoperative setting with the use of sedatives and hypothermia. Although the use of sedatives so far has not shown to change CO_2_‐induced CVR (Mariappan et al. 2014), it could minimize metabolism and consequently decreasing the rate of CO_2_ production. In addition to sedatives, slight hypothermia will also slow metabolism down, something that is done standardly for neurosurgical procedures. Finding a signal plateau only in the gray matter of the affected hemisphere may be explained by the model presented by Sobczyk et al. (for reference see figure 6 in Sobczyk et al. 2014). The gray matter of the affected hemisphere could be close to its full vasodilatory capacity and adding a vasoactive stimulus may result in only minor vasodilatation after which maximal dilatory capacity is reached. Increasing the vasoactive stimulus will not result in increased vasodilatation and therefore not in subsequent BOLD signal increase.

The absence of a plateau in the white matter can be explained by a slower dynamic vessel response as demonstrated in figure 4 of Bhogal et al. (2014) and a higher vascular resistance (van der Zwan et al. 1993).

### Frequency‐adjusted sine

A second improvement is the introduction of a sine model combining two different task frequencies, correcting for different durations of BH and resting periods. The need for frequency adjustment can be seen in the phase and coherence. A high number of responding voxels in the unaffected hemisphere showed a negative phase, confirming that the sine duration of 1 task frequency during BH period for healthy hemisphere is too long (Fig. 1). For both hemispheres the frequency‐adjusted sine provides a slightly better fit, which was significant for the unaffected hemisphere after difference analysis. Therefore, the frequency‐adjusted sine may result in a more sensitive and accurate model to differentiate healthy from unhealthy tissue. To better describe the pathological time series with a sine model, a future model needs to parameterize the response transformation between healthy and unhealthy tissue.

### Novel Optimal Signal Models

CVR only showed a significant hemispheric difference for the Unaffected Hemisphere, despite clear visual differences in the sagittal sinus. Sagittal Sinus had a high variation in CVR and therefore a positive difference was merely seen when analyzing the subtracted difference itself. For CVR, no significant difference between the models was found.

As for coherence, a well‐defined difference between both hemispheres for both models was shown. When comparing both models, coherence in both hemispheres of the Unaffected Hemisphere was significantly positively different from those of the sagittal sinus (Fig. 4). This may be a result of the Unaffected Hemisphere being a mix of positive and negative time series, thus averaging out its time series, whereas the sagittal sinus is a method only influenced by positive responding voxels and is therefore more representative of a “perfect” time series of that subject. Comparing voxels to a perfect time series or an averaged time series will result in distinct differences.

The new meaning of phase of the sagittal sinus model is interesting. Due to the set location of the sagittal sinus, the phase map of the sagittal sinus model is a combined arterial/venous phase map. Negative phase combines the venous temporal lag and part of the dynamic response, whereas positive phase consists of prolonged arterial temporal lag and the dynamic response. In contrast, the phase calculations with Unaffected Hemisphere result in a mixed arterial map. As the “Optimal Signal” models both result in different maps, we cannot compare the phase of Optimal Signal models neither to each other nor to the phase of the sine models.

Optimal Signal regressors can be very useful tools for standardization of BH analysis. Even after proper instruction and training, a high number of subjects do not perform the given BH paradigm correctly (van Beek et al. 2011). Optimal Signal models are independent of compliance and therefore CVR maps will be much more robust for uncompliant patients. Moreover, the CVR is independent of the scaling and dynamic range of the images, which allows the comparison of CVR maps obtained on MR systems of different vendors, and also after a recalibration of the system. It might also be (more) independent of the metabolic rate of the subject, cardiac capabilities and rate adaptability of the subject, pulmonary state, and peripheral CVR of the extremities. Besides, as these models are not limited to BH studies, these methods could potentially be very useful as a tool to investigate CVR between different vasoactive stimuli or between different scanners with a similar vasoactive stimulus.

These “optimal Signal” models are also able to determine the PaCO_2_ evolution during the BH, what cannot be done with CO_2_ tracing during BH.

### Limitations

All proposed analysis regressors used in this study are only surrogate time courses for CO_2_‐dependent changes (in the absence of continuous CO_2_ tracing), therefore resulting in semiquantitative % BOLD signal changes. This limits the reproducibility and increases intra‐ and intersubject variability (Bright and Murphy 2013). Introducing computer‐paced breathing (Scouten and Schwarzbauer 2008), adding a respiratory belt to correct for inspiratory depth, and compliance (Thomason and Glover 2008), or using expiratory BH to avoid the biphasic response of inspiratory BH (Kastrup et al. 1999; Scouten and Schwarzbauer 2008) may improve reproducibility. However, the best way to increase reproducibility is to extent the duration of BH, by aiming for a more robust CO_2_ increase (Lui et al. 2002; Magon et al. 2009; Bright and Murphy 2013). Others have sought to decrease the variability by tracing PETCO_2_. In end effect, BOLD changes are strongly related to the increase in the PaCO_2_. However, the existence of a (patho)physiological gradient between PETCO_2_ and the PaCO_2_ can result in unreliable readings (Fierstra et al. 2013). Besides, BH will result in reduced arterial partial pressure of O_2_ (Sasse et al. 1996). When hypoxia is reached, reduced venous saturation and PaO_2_ will cause vasodilatation and result in an increase in CBF (Poulin et al. 1996; Bulte et al. 2012). However, hypoxic ranges of 60–80 mmHg PaO_2_ need to be reached to induce CBF changes, which is even with our long BH duration unlikely (Brugniaux et al. 2007; Willie et al. 2012). Furthermore, uncalibrated gas lines will further influence the accuracy and reproducibility of end tidal CO_2_ trace measurements. Sine models are very dependent on correct compliance. Our compliance was based on proper instruction of the anesthetic team to ensure a precise protocol execution. CVR measurements in CSF could be another indicator of the robustness of the BOLD‐MRI CVR test. Thomas et al. (2013) showed that an adequate CO_2_ stimulus will result in negative CVR due to high signal CSF displacement after a vasodilatory response. We also found negative CSF‐CVR in all our subjects (−0.10 ± 0.11).

A limitation of determining phase with maximum Pearson correlation coefficient is the need for range restraints. No literature exists on the most optimal ranges for any given model or BH paradigm. For instance, without restraints phase larger than 1 BH cycle can be found resulting in a reverse CVR. On the other hand, a phase range, too small to compensate for both temporal delay and dynamic response, will result in a suboptimal fit. Moreover, problems can arise when correlating one's model to a very limited noisy % signal change. Besides, if an MRI artifact like a spike occurs, maximum correlation will try to fit the regressor to the artifact, resulting in an incorrect phase. We corrected for possible artifacts and noise in our data by using novel advanced spatiotemporal filtering and smoothing.

Both the Unaffected Hemisphere and sagittal sinus models have some limitations. The cerebrovascular hemodynamic status of the unaffected hemisphere has high impact on the overall calculations of the Unaffected Hemisphere. With increasing BOLD time series differences within the hemisphere, the use of the unaffected hemisphere gets increasingly biased. Therefore, the mean coherence in the unaffected hemisphere with the Unaffected Hemisphere model does not approach “1” (i.e., maximal coherence), but a high number of voxels do (Fig. 3). This entails that voxels with a high coherence do not represent the most physiological healthy voxels, but voxels with time series most comparable to the average unaffected hemisphere time series. The physiological state of these voxels depends on the amount of impairment in the unaffected hemisphere. Besides, unilateral cerebrovascular pathologies could result in a more global (bihemispheric) diseased brain, thereby also exhibiting impairment in the unaffected hemisphere (Sam et al. 2014). Also bilateral disease may result in bilateral impaired CVR, thereby impeding selection of an unaffected hemisphere. As an example, we present a dataset of a subject with bilateral cerebrovascular disease in the [Link].

On the other hand, determining the correct location and the amount of voxels necessary for the optimal signal is a limitation for using the sagittal sinus model. As mentioned above, only the sagittal sinus model did not show a significant difference in CVR between both hemispheres. Despite clear visual differences (Fig. 2D) the high variations in CVR between the subjects resulted in a nonsignificant difference, which was only made clear after a difference analysis. This could be caused by different locations of masking, slight differences in number of voxels taken into account, which alters the extent of the signal, and additional unknown factors influencing the signal in the sagittal sinus.

The seemingly best location to place the mask is either the confluence of sinuses or at the conjunction of the superior sagittal sinus and the transverse sinus. Anatomy can differ vastly between subjects (Park et al. 2008) making it challenging to create a similar mask across subjects. This problem can be solved by normalization of the brain into MNI space, after which, one sagittal sinus mask can be used. Further evaluation of the BOLD signal of the sagittal sinus is necessary for deeper understanding of its properties and value for CVR analysis.

## Conclusions

Here, we present novel analysis methods derived from BOLD fMRI breath hold datasets for more exact CVR interpretation. First, adding a voxel‐wise phase to the BH‐based sine model results in better differentiation between affected (i.e. CVR impairment) and unaffected brain tissue. Furthermore, combining the two task frequencies results in increased coherence in the unaffected hemisphere, which supports the use of the frequency‐adjusted sine. Optimal Signal models provide a feasible alternative to the voxel‐wise sine models where the sagittal sinus may be advantageous over the unaffected hemisphere, as it is independent of hemispheric CVR impairment.

## Conflict of Interest

None of the authors expresses a conflict of interest related to this study, its design, results, and conclusions.

## Appendix

Time series, CVR, and coherence of a subject with bilateral internal carotid occlusion and left vertebral occlusion treated with left EC‐IC bypass revascularization. Both hemispheres are taken separately as “unaffected” hemisphere. The sagittal sinus is taken as a comparison. Color scales are presented below the images. Time series are presented within −2 and 2%. CVR ranges between −1 and 1% BOLD signal change/% signal change.

This figure illustrates why the hemodynamic state of a single hemisphere can confound CVR and coherence interpretation within the unaffected hemisphere model. Due to large differences in time series within both hemispheres, the range of the time series of the hemisphere are limited when compared with the time series of the sagittal sinus. These similar time series result in a high overall CVR for both hemispheres (i.e., more % signal change per % change of model). Moreover, by using the unaffected hemisphere model, evaluation of coherence is challenging due to the unknown extent of CVR impairment in the chosen hemisphere. For instance, the EC‐IC bypass revascularization resulted in more positive responding voxels in the left hemisphere. This in turn results in a slightly better defined time series, and hence increased range of coherence, resulting in better differentiation between impaired and nonimpaired voxels. The right hemisphere is an average of voxels reacting to a lesser extent. Coherence over the brain, even in parts with supposedly less impaired voxels coherence is low.

The overall coherence of the sagittal sinus is higher compared to both models and areas showing high and low coherence in the left unaffected hemisphere are more pronounced.
